# Evaluation of surgical approach and adjuvant therapy in uterine sarcomas: an 11-year population-based study

**DOI:** 10.1186/s12885-026-16136-6

**Published:** 2026-07-23

**Authors:** Filip Herbst, Erik Holmberg, Louise Moberg, Christer Borgfeldt

**Affiliations:** 1 Department of Obstetrics and Gynecology, Skåne University Hospital Lund, Lund University, Klinikgatan 12, Lund, 221 85 Sweden; 2 Department of Oncology, Institute of Clinical Sciences, University of Gothenburg, Gothenburg, Sweden; 3 Department of Obstetrics and Gynecology, Linköping University Hospital, Linköping University, Linköping, Sweden

**Keywords:** Uterine sarcoma, Leiomyosarcoma, Overall survival, Treatment, Minimally invasive surgery, Chemotherapy

## Abstract

**Background:**

Uterine sarcomas are rare and high-quality evidence regarding optimal treatment is limited and difficult to produce.

**Methods:**

A total of 618 patients with uterine sarcoma were identified in the Swedish Quality Registry for Gynecologic Cancer (SQRGC) from 2010 to 2020. Survival was analyzed according to stage, histology and treatment modality.

**Results:**

The incidence of uterine sarcoma was 1.43 per 100,000 woman-years. Overall survival after 1 and 5 years was 75.9% and 44.1% respectively, with survival primarily influenced by stage, age and histological type. Adjuvant chemotherapy was not associated with improved survival, neither in localized nor advanced disease. Among patients with stage I disease, overall survival was higher after minimally invasive surgery (MIS) 81%, compared to open surgery 57% (*p* = 0.007); however, after entropy balancing no significant difference was observed.

**Conclusions:**

MIS showed comparable survival to open surgery in early-stage uterine sarcomas, with a non-significant trend toward improved survival. Entropy balancing supported this finding but residual confounding and selection bias cannot be excluded due to the observational design. Adjuvant chemotherapy was not associated with improved survival in uterine sarcoma patients overall or in leiomyosarcomas specifically, neither in localized nor advanced disease.

**Supplementary Information:**

The online version contains supplementary material available at 10.1186/s12885-026-16136-6.

## Highlights

Adjuvant chemotherapy shows no survival benefit in uterine sarcoma patients, neither in localized nor spread disease.

Minimally invasive surgery showed comparable survival to open surgery in early-stage uterine sarcomas.

Overall survival in uterine sarcoma patients remains poor but is highly affected by histological type, stage and age.

**Supplementary Information:**

The online version contains supplementary material available at 10.1186/s12885-026-16136-6.

## Background

Uterine sarcomas account for 3–5% of malignant uterine disease, and about 7% of soft tissue sarcomas [1–3]. Despite their rarity, these tumors are associated with a disproportionately high mortality, underscoring the need for optimized diagnostic and therapeutic strategies.

Uterine sarcomas can be divided into five main histological types; leiomyosarcoma (LMS), low-grade endometrial stromal sarcoma (lgESS), high-grade endometrial stromal sarcoma (hgESS), adenosarcoma (AS) and undifferentiated uterine sarcomas [1, 3, 4]. Mortality differs substantially between these groups with lgESS being the most favorable [1]. Carcinosarcomas, previously categorized as sarcomas, are now considered endometrial cancers and are not included in this paper. Prognosis is also highly affected by FIGO stage. Since 2009, FIGO uses two slightly different staging systems, one for LMS, endometrial stromal sarcomas and undifferentiated sarcomas and one for AS [3–5].

Treatment of uterine sarcomas is mainly based on surgery with total hysterectomy and in advanced stages complete removal of all macroscopic tumor [3, 4]. Bilateral oophorectomy is usually considered standard treatment even though survival benefit has not been proven [3, 6]. Lymphadenectomy is not recommended, except in cases with enlarged lymph nodes, since it does not improve survival [7–9]. In recent years, minimally invasive surgery (MIS) has gained traction in gynecologic oncology due to its association with reduced perioperative morbidity and faster recovery [10]. There is a lack of data regarding the safety of a minimally invasive surgical approach and current recommendations include only to consider this in cases where the integrity of the uterus can be assured without spillage of uterine tissue [4].

The role of adjuvant therapies, including chemotherapy and radiotherapy, remains controversial and is often guided by limited evidence and institutional preferences [11]. Recommendations vary depending on stage and histology. Chemotherapy is generally not recommended in early disease since no survival benefit has been shown [9, 12]. Adjuvant endocrine therapy is often recommended in advanced and recurrent lgESS [4, 7].

This study aims to evaluate survival outcomes in patients with uterine sarcoma in Sweden between 2010 and 2020, with particular focus on the impact of surgical approach and adjuvant chemotherapy. Using comprehensive data from the Swedish Quality Registry for Gynecologic Cancer (SQRGC), the study seeks to clarify the prognostic significance of these treatment modalities and to provide real-world evidence to support clinical decision-making in this rare and heterogeneous group of malignancies.

## Methods

The Swedish Quality Registry for Gynecologic Cancer (SQRGC) has prospectively collected nationwide data on uterine cancers since 2010. The reporting is separate from gynecologists, oncologists and pathologists and includes patient information, tumor characteristics, treatment and follow-up. SQRGC receives information about deaths through linkage to the Swedish Population Register. The completeness of the SQRGC has been shown to be 96% for uterine malignancies reported to the National Cancer Registry [13]. All women ≥ 18 years with histologically verified uterine sarcoma diagnosed between January 1st 2010 and December 31st 2020 were identified in the SQRGC. One woman had two different diagnoses at the same date (LMS and hgESS). The diagnosis of LMS was chosen for further analysis and the woman was included only once. Follow-up was continued until death, emigration or June 23, 2022 when data was retrieved. Diagnosis of uterine sarcomas in Sweden is made according to the WHO classification of tumors. In the SQRGC uterine sarcomas are grouped in five categories; leiomyosarcoma, low-grade stromal sarcoma, adenosarcoma, high-grade stromal sarcoma and sarcoma NOS (not otherwise specified). Sarcoma NOS includes undifferentiated sarcomas and rare types of sarcomas. 

### Statistics

Incidence was calculated as crude incidence in women ≥ 18 years old, and as age standardized incidence according to European standard population, to facilitate international comparisons [14]. Survival time was defined as the interval from diagnosis to death, emigration, or June 23, 2022, whichever occurred first. Overall survival (OS) and relative survival (RS) were estimated with 95% confidence intervals (CI). OS was calculated using Kaplan–Meier estimates [15]. Hazard ratios (HR) were obtained from Cox proportional hazards regression models [16]. The proportional hazards assumption was assessed using Schoenfeld residuals [17]. RS, interpreted as a measure of net survival (i.e., survival from the disease of interest in the absence of other causes of death), was estimated using the Pohar–Perme estimator [18] implemented in Stata via the stns command [19]. Expected survival, stratified by sex, attained age, and calendar year, was derived from national life tables provided by Statistics Sweden (Statistiska centralbyrån, SCB). For comparison of surgical techniques MIS was defined as laparoscopic, robot-assisted laparoscopic or vaginal surgery and only the technique used at the primary operation was taken into account. We estimated the effect of MIS versus open surgery on time-to-event outcomes using a weighted Cox proportional hazards model targeting the Average Treatment effect on the Treated (ATT). To create a pseudo-control group comparable to MIS patients, we applied entropy balancing [20] to reweight patients treated with open surgery so that the weighted distribution of baseline covariates matched that of the MIS group (effect among MIS patients). Open-surgery patients were reweighted using entropy balancing so that the weighted means of standardized age (zAge) and its square (zAge²), thereby aligning both the mean and variance of Age, matched those of the MIS group, together with the proportions of morphology (leiomyosarcoma vs. other), chemotherapy (no vs. yes) and stage IA vs. IB. Age was standardized for balancing but entered unstandardized and linearly in the Cox model. Models used probability weights with robust (Huber–White) standard errors clustered by patient ID and the Breslow method for ties. Balance was evaluated via standardized mean differences (SMD). Weight diagnostics included the effective sample size, 95th percentile (P95), and maximum weight. Proportional-hazards (PH) diagnostics used Schoenfeld-type tests; the global test did not reject PH (χ²=6.24, df = 4, *p* = 0.182), with a small signal for morphology (*p* = 0.035). All statistical analyses were performed in Stata (version StataNow/MP 19.5 for Mac, StataCorp, College Station, TX). A two-sided p-value < 0.05 was considered statistically significant. No imputation of missing data has been done.

## Results

In the SQRGC there were 618 registered cases of uterine sarcoma in Sweden from 2010 to 2020, with an average of 56 cases per year (Table 1). Crude incidence was 1.43 cases per 100,000 women-years and the age-standardized incidence was 1.16 cases per 100,000 women-years (95%CI: 1.06–1.26). The mean age at diagnosis was 62.5 years (range: 24–100). LgESS affected the youngest women at an average age of only 55.6 years and sarcoma NOS, including undifferentiated sarcomas, affected the oldest patients at an average of 73.0 years old. The most common histological type was LMS, accounting for 322 cases (52.1%). Total median follow-up time was 2.7 years, and median follow-up time with other endpoints than death was 5.6 years.

**Table 1:** Study cohort by morphology

	Stromal sarcoma	Adenosarcoma	Leiomyosarcoma	Stromal sarcoma	Sarcoma NOS	Total
	Low-grade			High-grade		
	*N* = 89	*N* = 70	*N* = 322	*N* = 95	*N* = 42	*N* = 618
Age, mean (SD)	55.6 (15.9)	67.2 (15.0)	61.1 (12.6)	65.5 (13.9)	73.0 (11.3)	62.5 (14.2)
Age, median (IQR)	52.0 (45.0–69.0)	69.0 (58.0–79.0)	61.5 (52.0–70.0)	67.0 (54.0–76.0)	74.0 (65.0–80.0)	63.0 (52.0–74.0)
Age group						
<50	36 (40.4%)	8 (11.4%)	68 (21.1%)	13 (13.7%)	0 ( 0.0%)	125 (20.2%)
50–59	22 (24.7%)	12 (17.1%)	82 (25.5%)	20 (21.1%)	5 (11.9%)	141 (22.8%)
60–69	10 (11.2%)	16 (22.9%)	88 (27.3%)	19 (20.0%)	12 (28.6%)	145 (23.5%)
70–79	11 (12.4%)	18 (25.7%)	64 (19.9%)	28 (29.5%)	13 (31.0%)	134 (21.7%)
>79	10 (11.2%)	16 (22.9%)	20 ( 6.2%)	15 (15.8%)	12 (28.6%)	73 (11.8%)
FIGO stage						
I	59 (66.3%)	62 (88.6%)	183 (56.8%)	48 (50.5%)	8 (19.0%)	360 (58.3%)
IA	24 (27.0%)	37 (52.9%)	34 (10.6%)	15 (15.8%)	2 ( 4.8%)	112 (18.1%)
IB	27 (30.3%)	19 (27.1%)	140 (43.5%)	31 (32.6%)	5 (11.9%)	222 (35.9%)
IC, IX	8 ( 9.0%)	6 ( 8.6%)	9 ( 2.8%)	2 ( 2.1%)	1 ( 2.4%)	26 ( 4.2%)
II	7 ( 7.9%)	3 ( 4.3%)	26 ( 8.1%)	8 ( 8.4%)	4 ( 9.5%)	48 ( 7.8%)
III	10 (11.2%)	1 ( 1.4%)	19 ( 5.9%)	15 (15.8%)	5 (11.9%)	50 ( 8.1%)
IV	4 ( 4.5%)	2 ( 2.9%)	68 (21.1%)	14 (14.7%)	10 (23.8%)	98 (15.9%)
Missing	9 (10.1%)	2 ( 2.9%)	26 ( 8.1%)	10 (10.5%)	15 (35.7%)	62 (10.0%)
Treatment						
Surgery only	41 (46.1%)	46 (65.7%)	139 (43.2%)	26 (27.4%)	10 (23.8%)	262 (42.4%)
Surgery+chemotherapy	3 ( 3.4%)	6 ( 8.6%)	80 (24.8%)	18 (18.9%)	10 (23.8%)	117 (18.9%)
Radiotherapy/radiochemotherapy +/- surgery	5 ( 5.6%)	4 ( 5.7%)	8 ( 2.5%)	7 ( 7.4%)	1 ( 2.4%)	25 ( 4.0%)
Surgery+endochrine therapy	9 (10.1%)	0 ( 0.0%)	1 ( 0.3%)	3 ( 3.2%)	0 ( 0.0%)	13 ( 2.1%)
Other treatment combinations	3 ( 3.4%)	0 ( 0.0%)	16 ( 5.0%)	11 (11.6%)	3 ( 7.1%)	33 ( 5.3%)
No treatment	3 ( 3.4%)	2 ( 2.9%)	9 ( 2.8%)	7 ( 7.4%)	12 (28.6%)	33 ( 5.3%)
No information	25 (28.1%)	12 (17.1%)	69 (21.4%)	23 (24.2%)	6 (14.3%)	135 (21.8%)

**Fig. 1:**
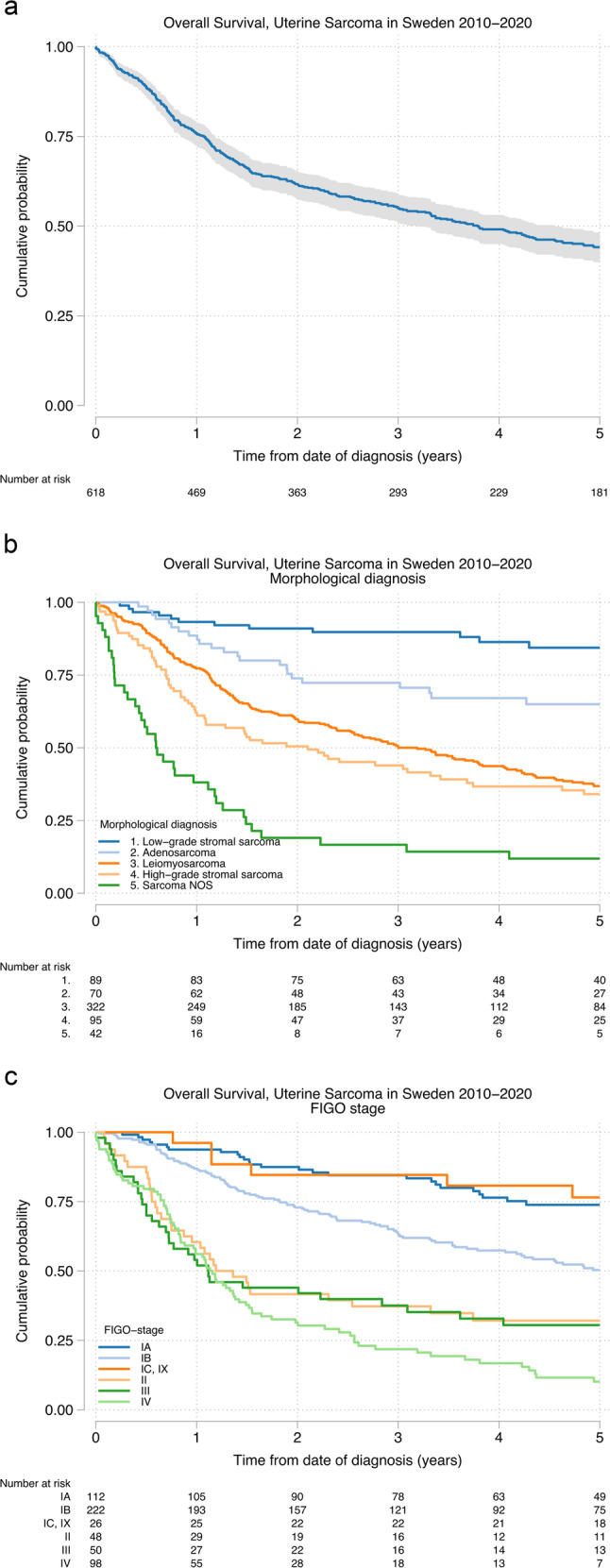
Overall survival for all patients with uterine sarcoma (**a**) (grey area shows 95% CI), separated by histological type (**b**) and separated by FIGO stage (**c**)

Overall survival after one year was 75.9% (95%CI: 72.3–79.1) and after five years 44.1% (95%CI: 39.9–48.3) (Fig. 1a). RS was similar with a total 1- and 5-year relative survival of 76.9% and 47.8% respectively. Survival differed substantially according to histological type (Fig. 1b). LgESS had the highest 5-year OS (84.4%) (95%CI: 73.7–91.0) followed by AS (65.0%) (95%CI: 51.9–75.3). LMS (36.8%) (95%CI:31.3–42.4) and hgESS (34.1%) (95%CI:24.5–43.9) had worse survival and sarcoma NOS (11.9%) (95%CI: 4.4–23.6) the worst.

FIGO stage I was most common, 64.7% of staged cases (missing stage *n* = 62) (Table 1). The second most common stage was IV (17.6%) followed by III (9.0%) and II (8.6%). Stage greatly influenced survival with stage IA, IC and IX (stage I not further specified) having a 5-year survival of approximately 75% but stage IB having a 5-year survival of 50.2%, stage II and III 32.2% and 30.5% respectively and stage IV only 10.2% (Fig. 1c).

**Fig. 2:**
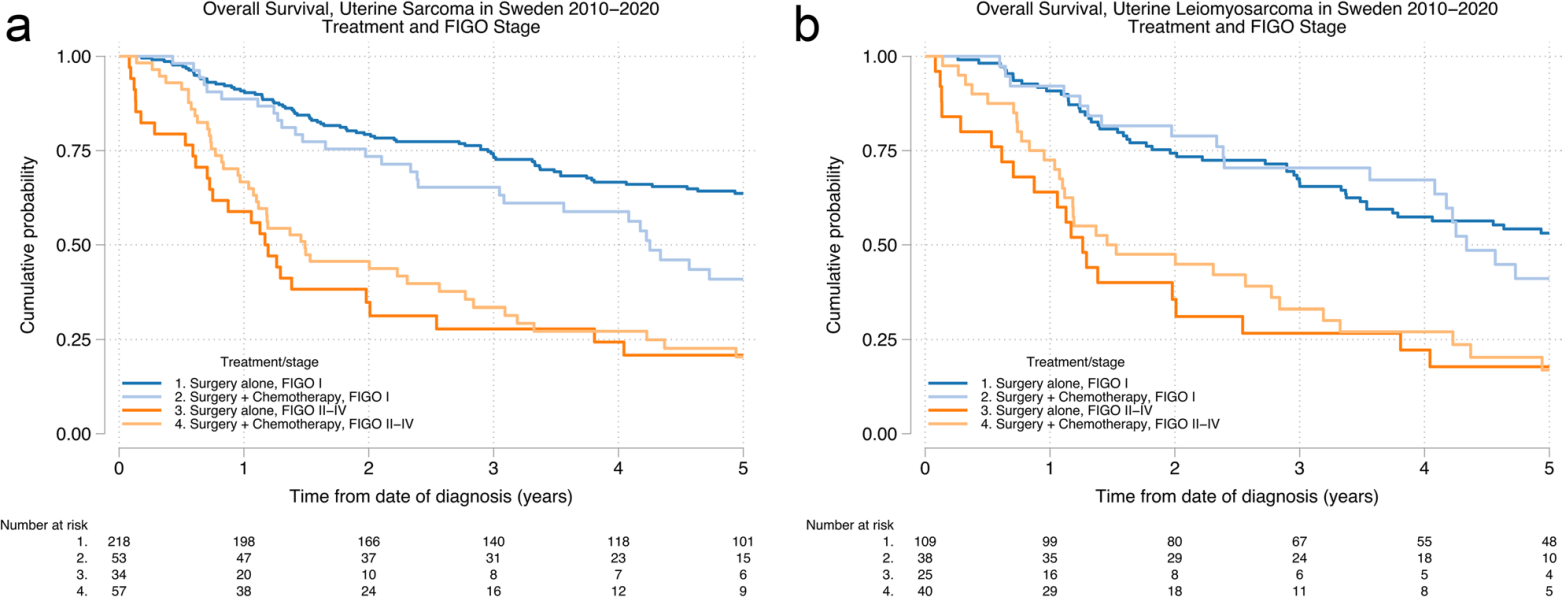
Overall survival after stratification for FIGO stage and treatment, for all uterine sarcomas, n362 (**a**), and for leiomyosarcomas separately, n212 (**b**)

The most given treatments were surgery only (42.4% of all, 54.2% of cases with treatment registered, missing *n* = 135), followed by surgery and chemotherapy (18.9% of all, 24.2% of registered) (Table 1). In a sub analysis of patients with LMS there was no improved survival from added chemotherapy (Fig. 2b). The Cox proportional hazards regression analysis for patients with LMS showed a significant negative effect of increasing age on OS and no difference in OS when comparing surgery only to surgery and chemotherapy, neither in stage I (HR 1.17, 95%CI 0.69–1.98) nor in stage II-IV (HR 0.87, 95%CI: 0.47–1.61) (Table 2). Only 4% of the patients had radiotherapy or radio-chemotherapy in addition to surgery and 2.1% had endocrine therapy in addition to surgery. For 5.3% of the patients no treatment was given. This group includes patients in a condition to poor to tolerate treatment (*n* = 17), patients with comorbidities preventing treatment (*n* = 4), patients that died before initiation of treatment (*n* = 7) and patients who declined treatment (*n* = 5).

**Table 2:** Univariable and multivariable Cox proportional hazards regression analysis for mortality (yes/no) for women with leiomyosarcoma (surgery only or surgery + chemotherapy)

**Leiomyosarcoma all** **FIGO stages,** ***n*** **= 212**	**Univariable model** ^**a**^ **HR (95% CI),** ***P***	**Multivariable model 1** ^**b**^ **HR (95% CI),** ***P***	**Multivariable model 2** ^**c**^ **HR (95% CI),** ***P***
Age, 10-year increments	1.32 (1.13–1.55), 0.001	1.25 (1.07–1.46), 0.005	1.26 (1.07–1.48), 0.005
FIGO stage			
I	Reference	Reference	Reference
II-IV	2.90 (2.01–4.19), < 0.001	2.71 (1.87–3.92), < 0.001	2.60 (1.75–3.87), < 0.001
Treatment modality			
Surgery only	Reference	-	Reference
Surgery + chemotherapy	1.43 (0.99–2.06), 0.054	-	1.11 (0.75–1.66), 0.593
***Leiomyosarcoma*** ***FIGO stage I, n = 147***	***Univariable model*** ^**a**^ ***HR (95% CI), P***	***Multivariable model 3*** ^***d***^ ***HR (95% CI), P***	
Age, 10-year increments	1.47 (1.18–1.83), 0.001	1.47 (1.18–1.84), 0.001	
Treatment modality			
Surgery only	Reference	Reference	
Surgery + chemotherapy	1.14 (0.67–1.94), 0.628	1.17 (0.69–1.98), 0.570	
***Leiomyosarcoma*** ***FIGO stage II - IV, n = 65***	***Univariable model*** ^***a***^ ***HR (95% CI), P***	***Multivariable model 3*** ^***d***^ ***HR (95% CI), P***	
Age, 10-year increments	1.06 (0.85–1.33), 0.578	1.04 (0.82–1.33), 0.736	
Treatment modality			
Surgery only	Reference	Reference	
Surgery + chemotherapy	0.84 (0.48–1.47), 0.532	0.87 (0.47–1.61), 0.662	
***Leiomyosarcoma*** ***FIGO stage I, n = 121***	***Univariable model*** ^***a***^ ***HR (95% CI), P***	***Multivariable model 4*** ^***e***^ ***HR (95% CI), P***	
Age, 10-year increments	1.47 (1.18–1.83), 0.001	1.56 (1.18–2.06), 0.002	
Surgery method			
Minimally invasive surgery (MIS)	Reference	Reference	
Open surgery	1.19 (0.51–2.79), 0.687	1.38 (0.58–3.26), 0.468	

^a^Univariable Cox proportional hazards regression analysis with hazard ratio (HR), 95% confidence interval (CI) and *p*-value

^b^Multivariable model 1 includes age and FIGO stage in the Cox proportional hazards regression analysis with HR (95% CI) and *p*-value

^c^Multivariable model 2 includes age, FIGO stage and treatment modality in the Cox proportional hazards regression analysis with HR (95% CI) and *p*-value

^d^Multivariable model 3 includes age and treatment modality in the Cox proportional hazards regression analysis with HR (95% CI) and *p*-value

^e^Multivariable model 3 includes age and surgical method in the Cox proportional hazards regression analysis with HR (95% CI) and *p*-value

**Fig. 3:**
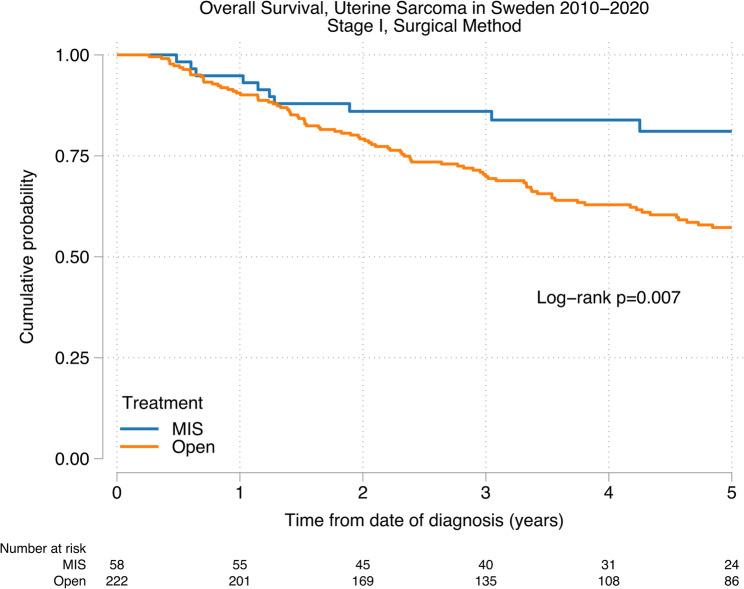
Overall survival for patients with uterine sarcoma, FIGO stage I, separated by surgical method

The most common primary surgical method was open surgery (*n* = 341) followed by robot-assisted laparoscopic surgery (*n* = 44), traditional laparoscopic surgery (*n* = 13) and vaginal surgery (*n* = 12). Five-year survival for stage I, all morphologies, with open surgery was 57.2% (95% CI: 50.0–63.9) and with MIS 81.1% (95% CI: 67.3–89.5), *p* = 0.007 (Fig. 3). When analyzing the 220 FIGO stage IA-IB patients treated with surgery only or surgery + chemotherapy. Pre-weighting imbalances were present (e.g., age SMD − 0.18; morphology + 0.24). Entropy balancing achieved near-perfect balance (supplement 1). Weights were well-behaved (effective sample size = 158.8; max = 1.01; P95 = 0.37). In the weighted Cox model, MIS showed a non-significant trend of lower hazard than open surgery (HR 0.56, 95% CI 0.31–1.02; *p* = 0.059) (Table 3). Older age increased hazard (per 10 year HR 1.63, 95% CI 1.27–2.08; *p* < 0.001). Surgery+chemotherapy had a higher hazard than surgery only (HR 1.76, 95% CI 1.01–3.08; *p* = 0.047). The 5-year survival for stage I LMS with primary open surgery was 53.1% (95%CI: 43.3–62.0), and with MIS 69.2% (95%CI:45.6–84.2), with a HR of 0.84 (95%CI: 0.36–1.97) for MIS compared to open surgery. When adjusting for age the HR was 0.73 (95%CI: 0.31–1.72) (Table 2). Thirty-two patients had more than one registered surgery, and one patient had more than two surgeries (four surgeries). Of the 32 patients that had repeated surgery, 13 had it within 6 months. Of these 13 patients 8 had initial open surgery, 3 had vaginal surgery and 2 had laparoscopic surgery. Of the 19 patients who had repeated surgery > 6 months from the initial surgery 16 had initial open surgery, 2 had laparoscopic and 1 had vaginal surgery.

**Table 3:** Univariable and multivariable Cox proportional hazards regression analyses for mortality using entropy balancing in FIGO stage IA-IB uterine sarcoma (*N* = 220)

Variables	Univariable HR (95% CI)	*P*	Multivariable HR (95% CI)	*P*
Surgery method				
Open surgery	1.0		1.0	
Minimal invasive surgery (MIS)	0.62 (0.32–1.22)	0.165	0.56 (0.31–1.02)	0.059
Age, 10-year increments	1.39 (1.15–1.69)	**0.001**	1.63 (1.27–2.08)	**< 0.001**
FIGO stage				
IA	1.0		1.0	
IB	3.03 (1.15–5.69)	**0.001**	3.42 (1.51–7.74)	**0.003**
Morphology				
Leiomyosarcoma	1.0		1.0	
Other	0.46 (0.25–0.84)	**0.011**	0.73 (0.35–1.54)	0.410
Treatment modality				
Surgery only	1.0		1.0	
Surgery + chemotherapy	2.31 (1.25–4.27)	**0.007**	1.76 (1.01–3.08)	**0.047**

Univariable and multivariable Cox regression models for survival of the LMS patients showed independently significant increased risk of death in age groups separated in 10-year intervals, and Stage II-IV vs. I (Table 2). Surgery+chemotherapy vs. surgery only was not independently significant HR:1.11 (95% CI:0.75–1.66).

## Discussion

In this study, including all registered uterine sarcomas in Sweden during eleven years, no survival advantage was observed for patients treated with surgery and adjuvant chemotherapy compared to those who underwent only surgery. Since uterine sarcomas represent a highly heterogenous group of tumors, these findings have to be interpreted with caution, keeping in mind that chemotherapy was administered more frequently to patients with less favorable histological types. Leiomyosarcoma was the only subtype with a sufficient sample size for separate analysis. No benefit from added chemotherapy was found in LMS patients, neither in FIGO stage I nor Stage II-IV. These findings align with previous studies demonstrating similar or shorter overall and disease-free survival with chemotherapy in uterine sarcomas overall [21, 22], and no survival benefit from adjuvant chemotherapy in early-stage LMS [12, 23]. Data on the impact of chemotherapy in advanced uterine LMS are limited, but several regimens have shown promising responses [9]. The absence of survival benefit in our study may reflect the small number of advanced LMS patients (*n* = 65) or a true absence of effect. Most previous survival studies are also retrospective including all but one in the meta-analysis by Bogani et al. [12], with a potential of selection bias. The few and rather small prospective studies performed have failed to show improved progression free survival with adjuvant chemotherapy [23, 24]. Hensley et al. initiated a randomized control trial comparing gemcitabine plus docetaxel to observation after surgery for LMS, but the study was closed early due to accrual futility [25], highlighting the difficulty in generating high level evidence. When all histological subtypes were analyzed together, the addition of chemotherapy was associated with reduced survival in patients with FIGO stage I sarcomas. Patients with FIGO stage I who received adjuvant chemotherapy did not exhibit excess early mortality, as might be expected if chemotherapy toxicity was the main cause. Although late adverse outcomes, such as cardiotoxicity, can occur, these are less frequent than early toxicities. The timing of excess mortality therefore argues against chemotherapy as the main explanation, a conclusion supported by multivariable Cox regression analyses in LMS showing no significant survival difference with added chemotherapy. This finding therefore likely reflects selection bias rather than a true negative effect of chemotherapy. In patients with stage I non-LMS sarcomas, those receiving adjuvant chemotherapy had significantly worse survival than those who underwent surgery alone, again most likely reflecting selection bias, with chemotherapy preferentially given to patients with poorer prognostic factors, including unfavorable histology.

LMS accounted for 52.1% of uterine sarcomas in this study, a slightly lower proportion than previously reported [2, 5]. HgESS, comprised 15.4%. LgESS was the third most common group at 14.4%, and AS accounted for 11.3% which is slightly higher than previously described [2, 5, 26]. Sarcomas NOS including undifferentiated sarcomas and rare subtypes represented 6.8% of cases. Comparisons in distribution with previous studies are somewhat difficult because of changes made in classification. Survival rates varied significantly among different histological groups. LgESS exhibited the highest 5-year survival rate (84.4%) followed by AS (65.0%). LMS (36.8%) and hgESS (34.1%) had worse survival and sarcoma NOS (11.9%) the lowest. These findings align with previous research [2, 7].

Stage is a well-established prognostic factor in uterine sarcoma [5]. Nonetheless, this study found almost no difference in survival between FIGO stages II and III, whereas IB displayed a substantially worse prognosis than IA and IC. This may be explained by other differences such as uneven distribution of histological types. Stage IC is for example exclusive to AS, which has a more favorable prognosis. FIGO stage I predominated in this study making up 58.3% of the cases. Stage II, III and IV accounted for 7.8%, 8.1% and 15.9% respectively, with 10.0% lacking stage information. Compared to previous reports our cohort included more stage II cases [6].

Since only 5.2% (*n* = 32) of patients underwent multiple surgeries, and merely 2.1% (*n* = 13) within six months, drawing conclusions regarding the impact of initial surgical approach on the risk of requiring additional surgery is challenging. Although the low rate of repeated surgery is positive, there is a possibility of underreporting of initial surgeries of unexpected sarcomas from regional hospitals, potentially causing secondary surgeries to be reported as primary ones. This is a limitation that cannot be addressed with the available data. The 19 patients who had second surgery after more than six months were likely operated due to recurrence or suspected recurrence.

Survival was significantly better after MIS than after open surgery in stage I sarcomas overall, while no significant difference was observed in stage I LMS. Selection bias may have favored MIS, since a known malignancy before surgery is likely associated with open surgery, potentially impacting survival numbers. Tumor size within stage I is unlikely to explain this difference, since both stage IA and IB independently showed higher OS with MIS (supplement 2). The MIS group included a higher proportion of adenosarcoma and fewer LMS cases (29.1% and 34.7%, respectively) compared with the open surgery group (11.5% and 57.8%), as well as slightly younger patients and fewer cases receiving chemotherapy. These imbalances were addressed using entropy balancing, and multivariable Cox regression after weighting showed a non-significant trend toward a survival advantage after MIS. Since many early-stage uterine sarcomas are not known preoperatively, but thought to be benign leiomyomas, surgical approach is often chosen without consideration of a potential sarcoma. With this observational study design, unknown factors such as imaging findings, rapid tumor growth and preoperative suspicion of malignancy may have influenced both surgical approach and survival outcomes, and residual confounding cannot be excluded. Data on morcellation were unavailable; however, if morcellation influenced outcomes, this would likely bias the results in favor of open surgery, and therefore cannot account for the observed association between MIS and survival. There are a substantial number of patients suspected of having sarcoma due to rapid growth or appearance on ultrasound, MRI or PET-CT [4, 27]. In these cases, our findings suggest that a minimally invasive approach may be considered, provided morcellation is avoided [9]. In a previous retrospective study, with 36 patients with stage I LMS, patients who underwent laparoscopic hysterectomy were compared to those who underwent open surgery. A significantly higher rate of locoregional recurrence was observed in the laparoscopic group (78% vs. 33.3%, *p* = 0.04). However, the surgical approach did not result in significant differences in OS [28]. The risk of undetected sarcoma in believed myomas increases with age. The FDA recommends laparoscopic morcellation only in appropriately selected women using a tissue containment system during myomectomy or hysterectomy [29].

This study is nationwide and includes all the registered cases of uterine sarcoma in Sweden over 11 years, collected in the SQRGC with excellent coverage of 96–98% [13, 30]. However, the retrospective nature of the study and missing data, such as FIGO stage (10%) and treatment information (21.8%), introduces potential biases. In addition, potentially important confounders, such as exact tumor size (beyond what could be inferred from stage) and preoperative suspicion of malignancy, were not available in the registry and could therefore not be included in the statistical adjustment. With 618 cases in total, subgroup analyses must be cautiously interpreted, considering known confounders such as histological type and stage. Central pathologic analysis of the specimens was not performed, which could impact the accuracy of the final diagnosis in some cases. However, since the patients come from a clinically based nationwide cohort, the external validity of the findings is likely to be high.

## Conclusion

MIS showed comparable overall survival to open surgery in early-stage uterine sarcomas, with a non-significant trend toward improved survival. Adjuvant chemotherapy was not associated with improved survival, particularly in LMS patients. Due to the observational design of this study, causal interpretations should however be made with caution. Overall survival in patients with uterine sarcoma in Sweden remains poor and is influenced by histological type, stage and age.

## Supplementary Information

Supplementary Material 1. Supplement 1. Descriptive unweighted characteristics (1a) and weighted characteristics following entropy balancing (1b) in patients with FIGO stage IA-IB uterine sarcoma (N = 220) who underwent either minimally invasive surgery (MIS) or open surgery, with or without adjuvant chemotherapy.

Supplementary Material 2. Supplement 2. Overall survival for patients with uterine sarcoma, FIGO stage Ia (blue) and Ib (orange), each separated by surgical method.

## Data Availability

This study made use of several national registers and, owing to legal concerns, data cannot be made openly available. Further information regarding the health registries is available from the Swedish National Board of Health and Welfare (https://www.socialstyrelsen.se/en/statistics-and-data/registers/) and Statistics Sweden (https://www.Scb.Se/en/). The code used in the analysis can be provided upon reasonable request directed to author Erik Holmberg.

## Abbreviations

AS: Adenosarcoma
CI: Confidence Interval
lgESS: Low-grade endometrial stromal sarcoma
hgESS: High-grade endometrial stromal sarcoma
HR: Hazard ratio
LMS: Leiomyosarcoma
MIS: Minimally invasive surgery
NOS: Not otherwise specified
SQRGC: The Swedish Quality Registry for Gynecologic Cancer
