# Supplementary material for: Evaluation of surgical approach and adjuvant therapy in uterine sarcomas: an 11-year population-based study
Source: BMC Cancer. 2026 Jul 23;26:888. doi: 10.1186/s12885-026-16136-6 (PMC13393630; doi:10.1186/s12885-026-16136-6)
Supplement: Supplementary file 1 — Supplementary Material 1. Supplement 1. Descriptive unweighted characteristics (1a) and weighted characteristics following entropy balancing (1b) in patients with FIGO stage IA-IB uterine sarcoma (N = 220) who underwent either minimally invasive surgery (MIS) or open surgery, with or without adjuvant chemotherapy. [file 12885_2026_16136_MOESM1_ESM.docx]

| **Supplement 1a. Descriptive unweighted characteristics in patients with FIGO stage IA-IB uterine sarcoma (N = 220) who underwent either minimally invasive surgery (MIS) or open surgery, with or without adjuvant chemotherapy.** | | |
| --- | --- | --- |
|  | Surgery method | |
|  | Open surgery | MIS |
| N (%) | 180 (100%) | 40 (100%) |
| **Variables** |  |  |
| Age, mean (SD) | 62.0 (13.6) | 59.2 (16.7) |
| FIGO stage |  |  |
| IA | 52 (28.9) | 22 (55.0) |
| IB | 128 (71.1) | 18 (45.0) |
| Morphology |  |  |
| Leiomyosarcoma | 103 (57.2%) | 17 (42.5%) |
| Other | 77 (42.8%) | 23 (57.5%) |
| Chemotherapy |  |  |
| No chemotherapy | 135 (75.0%) | 31 (77.5%) |
| Chemotherapy | 45 (25.0%) | 9 (22.5%) |

| **Supplement 1b.** **Descriptive data following entropy balancing in patients with FIGO stage IA-IB uterine sarcoma (N = 220) who underwent either minimally invasive surgery (MIS) or open surgery, with or without adjuvant chemotherapy. Open surgery numbers are weighted from the entropy balancing.** | | |
| --- | --- | --- |
|  | Surgery method | |
|  | Open surgery | MIS |
| N (%) | 40.0 (100%) | 40 (100%) |
| **Variables** |  |  |
| Age, mean (SD) | 59.2 (16.6) | 59.2 (16.6) |
| FIGO stage |  |  |
| IA | 22.0 (55.0%) | 22 (55.0%) |
| IB | 18.0 (45.0%) | 18 (45.0%0.56) |
| Morphology |  |  |
| Leiomyosarcoma | 17.0 (42.5%) | 17 (42.5%) |
| Other | 23.0 (57.5%) | 23 (57.5%) |
| Chemotherapy |  |  |
| No chemotherapy | 31.0 (77.5%) | 31 (77.5%) |
| Chemotherapy | 9.0 (22.5%) | 9 (22.5%) |
